# Integrated network analysis to explore the key genes regulated by parathyroid hormone receptor 1 in osteosarcoma

**DOI:** 10.1186/s12957-017-1242-0

**Published:** 2017-09-21

**Authors:** Donghui Guan, Honglai Tian

**Affiliations:** Department of Orthopaedics, Affiliated Hospital of Shandong University of Traditional Chinese Medicine, No. 16369 Jinshi Road, Lixia District, Jinan City, Shandong 250014 China

**Keywords:** Osteosarcoma, Parathyroid hormone receptor 1, Differentially expressed genes, Protein-protein interaction network, Integrated network

## Abstract

**Background:**

As an invasive malignant tumor, osteosarcoma (OS) has high mortality. Parathyroid hormone receptor 1 (*PTHR1*) contributes to maintaining proliferation and undifferentiated state of OS. This study is designed to reveal the action mechanisms of *PTHR1* in OS.

**Methods:**

Microarray dataset GSE46861, which included six *PTHR1* knockdown OS samples and six control OS samples, was obtained from the Gene Expression Omnibus database. The differentially expressed genes (DEGs) were identified and then performed with enrichment analysis separately using the limma package and DAVID online tool. Then, protein-protein interaction (PPI) network and module analyses were conducted using Cytoscape software. Using the WebGestalt tool, microRNAs (miRNAs) were predicted for the DEGs involved in the PPI network. Following this, transcription factors (TFs) were predicted and an integrated network was constructed by Cytoscape software.

**Results:**

There were 871 DEGs in the *PTHR1* knockdown OS samples compared with the control OS samples. Besides, upregulated *ZFPM2* was involved in the miRNA-DEG regulatory network. Moreover, TF *LEF1* was predicted for the miRNA-DEG regulatory network of the downregulated genes. In addition, *LEF1*, *NR4A2*, *HAS2*, and *RHOC* had higher degrees in the integrated network.

**Conclusions:**

*ZFPM2*, *LEF1*, *NR4A2*, *HAS2*, and *RHOC* might be potential targets of *PTHR1* in OS.

## Background

As an invasive malignant tumor, osteosarcoma (OS) often occurs in tubular long bones [1]. OS is the most common type of primary bone cancer, which has high occurrence rate in children and teenagers [2]. OS has rapid growth, high metastatic potential, and local aggressiveness; thus, it can result in high mortality [3]. In childhood cancers, OS accounts for about 2.4% of all malignant cancers and is the eighth most common tumor [4]. Therefore, revealing the molecular mechanisms of OS and developing novel therapies are of great importance.

Via upregulating matrix metalloproteinase 2 (*MMP2*), astrocyte elevated gene 1 (*AEG1*) functions in OS progression can be used for predicting the progression and prognosis of the disease [5, 6]. A previous study reports that *miR-203* is a tumor suppressor by mediating RAB22A, member RAS oncogene family (*RAB22A*) expression, and has correlation with the progression and carcinogenesis of OS [7]. Through nuclear factor kappa B (NF-κB) signaling pathway and mitochondria pathway, the inhibitor of growth 4 (*ING4*) plays a suppressive role in OS progression and serves as a potential target for treating OS [8, 9]. *miR-24* inhibits the metastasis of OS via regulating activated Cdc42-associated kinase (*ACK1*) through AKT/MMP pathways, which may be applied for the diagnosis and therapy of OS [10]. However, the pathogenesis of OS has not been comprehensively revealed.

Parathyroid hormone receptor 1 (PTHR1) signaling plays a critical role in keeping proliferation and undifferentiated state of OS; thus, *PTHR1* suppression may be used to inhibit OS proliferation and promote differentiation [11, 12]. Overexpression of *PTHR1* may contribute to OS progression through affecting aggressive phenotype and microenvironment [13]. To explore the action mechanisms of *PTHR1* in OS, we downloaded the microarray dataset GSE46861 which included both *PTHR1* knockdown OS samples and control OS samples. Then, differentially expressed genes (DEGs) were identified and performed with enrichment analysis. In addition, protein-protein interaction (PPI) network and module analyses, as well as integrated network analysis, were conducted to further screen the key targets of *PTHR1* in OS.

## Methods

### Data source and data preprocessing

Microarray dataset GSE46861, which was sequenced on the platform of GPL6246 [MoGene-1_0-st] Affymetrix Mouse Gene 1.0 ST Array [transcript (gene) version], was obtained from the Gene Expression Omnibus (GEO, http://www.ncbi.nlm.nih.gov/geo/) database. GSE46861 included six *PTHR1* knockdown OS samples and six control OS samples. The raw data of GSE46861 was normalized using the Robust Multiarray Average (RMA) method [14] in the R package Affy (version: 1.52.0).

### DEG screening and hierarchical cluster analysis

After the data normalization, the DEGs between *PTHR1* knockdown OS samples and control OS samples were screened using the Linear Models for Microarray Analysis (limma, version: 3.30.3) [15] package in R. The |log_2_ fold change (FC)| > 0.58 and *p* value < 0.05 were considered as the thresholds. In addition, pheatmap package (version 1.0.8, https://cran.r-project.org/web/packages/pheatmap/index.html) in R was utilized to perform the hierarchical cluster analysis.

### Functional and pathway enrichment analysis

Gene Ontology (GO) database can annotate genes and gene products from molecular function (MF), biological process (BP), and cellular component (CCo) aspects [16]. The Kyoto Encyclopedia of Genes and Genomes (KEGG) database can be applied for revealing gene functions and connecting genomic information with functional information [17]. Using the Database for Annotation, Visualization and Integrated Discovery (DAVID, version: 6.8, parameter: Classification Stringency was set as Medium) online tool [18], GO functional and KEGG pathway enrichment analyses were carried out for the DEGs, with the threshold of *p* value < 0.05.

### PPI network and module analyses

Search Tool for the Retrieval of Interacting Genes (STRING, version: 10.0) database [19], which included PPI pairs of multiple organisms, was used to analyze the PPI pairs among the DEGs. The required confidence (combined score) > 0.4 was used as the cutoff criterion. Afterwards, PPI network was visualized using Cytoscape software [20]. Based on the CytoNCA plugin (version 2.1.6, parameter: without weight) [21] in Cytoscape software, topological property analysis was performed for the nodes in the PPI network. According to closeness centrality (CCe), betweenness centrality (BC), and degree centrality (DC) scores, the hub nodes [22] in the PPI network were selected. Moreover, the MCODE plugin (version 1.2) [23] in Cytoscape software was used for identifying the significant modules in the PPI network. In addition, enrichment analysis for the genes involved in the most significant module was conducted using the DAVID online tool [18].

### Integrated network analysis

Using WEB-based gene set analysis toolkit (WebGestalt) tool [24], miRNAs were predicted for the DEGs involved in the PPI network, with false discovery rate (FDR, that was adjusted *p* value) < 0.05 and number of target genes ≥ 5 as the thresholds. Based on the iRegulon plugin (version 1.3) [25] in Cytoscape software, transcription factors (TFs) were further predicted for the miRNA-DEG regulatory network. The Minimum NEScore > 3 and FDR on motif similarity < 0.001 were set as thresholds. Finally, the obtained transcription regulation relationships were merged into the miRNA-DEG regulatory network, and the integrated network was visualized by Cytoscape software [20].

## Results

### DEG analysis and hierarchical cluster analysis

**Fig. 1:**
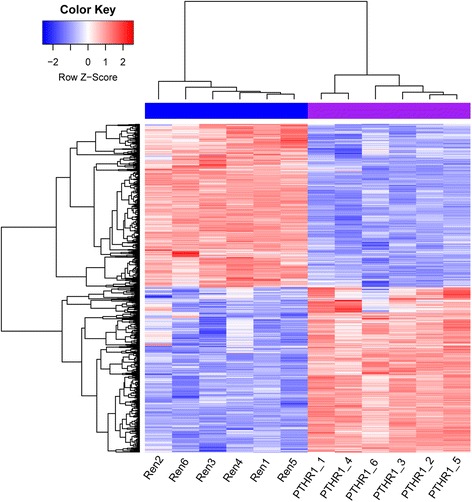
The heatmap of the differentially expressed genes by using pheatmap package

Compared with the control OS samples, there were a total of 871 DEGs (438 upregulated and 433 downregulated genes) in the *PTHR1* knockdown OS samples. The heatmap of the DEGs is shown in Fig. 1, which indicated that the expression of identified DEGs could correctly distinguish the two kinds of samples.

### Functional and pathway enrichment analysis

**Fig. 2:**
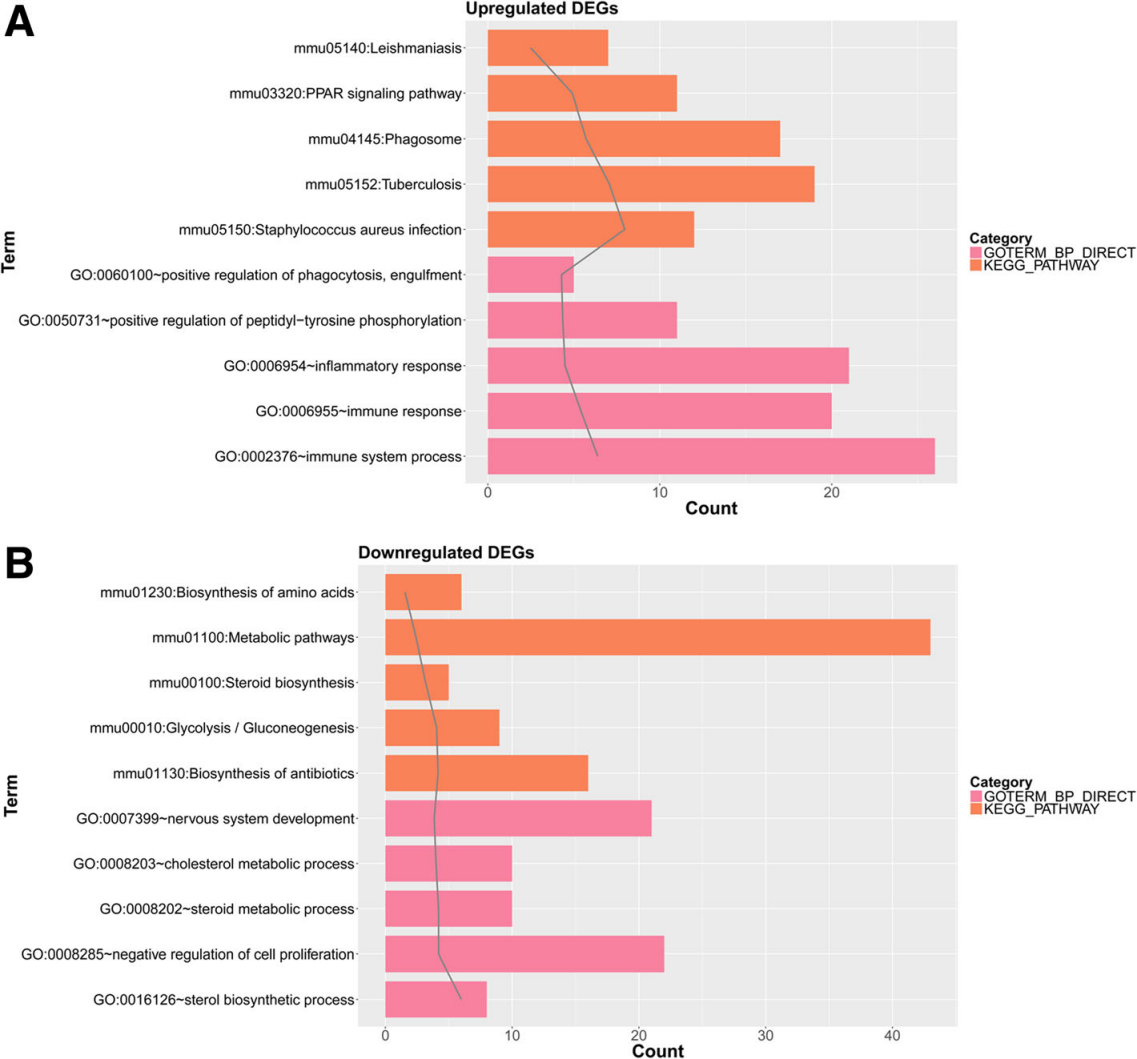
The top five GO_BP terms and pathways for the upregulated genes (**a**) and the downregulated genes (**b**), respectively, analyzed by Database for Annotation, Visualization and Integrated Discovery online tool. GO, Gene Ontology; BP, biological process; KEGG, Kyoto Encyclopedia of Genes and Genomes

Enrichment analysis was conducted for the upregulated genes and the downregulated genes, respectively. A total of 121 GO_BP terms and 20 pathways were enriched for the upregulated genes. The top five GO_BP terms and pathways are shown in Fig. 2a, mainly including immune system process (GO_BP, *p* value = 4.07E−07) and *Staphylococcus aureus* infection (pathway, *p* value = 1.09E−08). Besides, a total of 65 GO_BP terms and five pathways were enriched for the downregulated genes. Similarly, the top five GO_BP terms (such as sterol biosynthetic process, *p* value = 1.02E−06) and pathways (such as Biosynthesis of antibiotics, *p* value = 6.96E−05) are shown in Fig. 2b.

### PPI network and module analyses

**Fig. 3:**
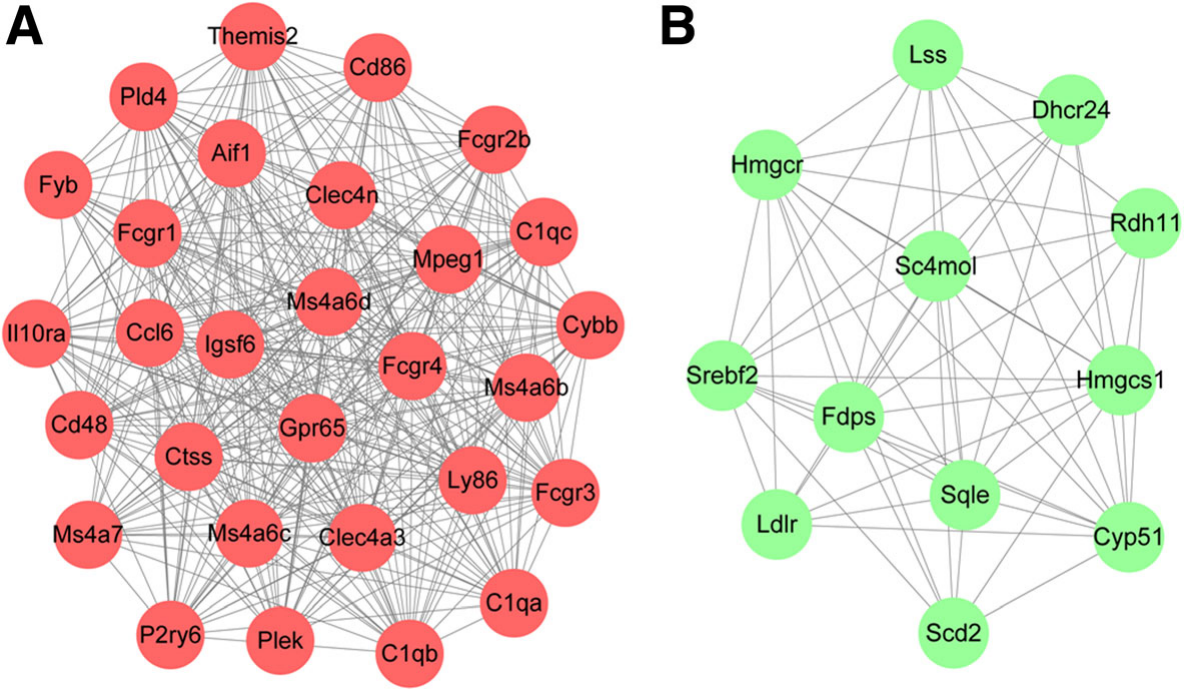
The most significant modules identified from the protein-protein interaction (PPI) networks for the upregulated genes (**a**) and the downregulated genes (**b**). The significant modules were analyzed by using MCODE plugin, and then module networks were visualized using Cytoscape software

The PPI network for the upregulated genes had 280 nodes and 1090 interactions. According to DC scores, the nodes with degrees larger than 30 are listed in Table 1. The most significant module (score = 25.786) identified from the PPI network for the upregulated genes had 29 nodes and 361 interactions (Fig. 3a). The upregulated genes involved in the most significant module were mainly enriched in immune system process (GO_BP, *p* value = 1.63E−07) and *Staphylococcus aureus* infection (pathway, *p* value = 2.59E−10) (Table 2 (A)).

**Table 1:** The nodes with degrees larger than 30 in the protein-protein interaction (PPI) network for the upregulated genes

Gene	Degree	Betweenness	Closeness
Ms4a6d	41.0	888.54470	0.055844676
Ly86	39.0	523.09924	0.055800000
Ms4a6b	39.0	2949.2993	0.055755395
C1qb	38.0	1383.42100	0.055766540
C1qa	38.0	3947.00420	0.056295400
Aif1	37.0	4212.39100	0.056261342
Mpeg1	37.0	323.91983	0.055655297
Fcgr1	37.0	501.60864	0.055633100
Ctss	37.0	640.59326	0.055588763
C1qc	37.0	749.12880	0.055811163
Clec4a3	36.0	475.66116	0.055710863
Gpr65	36.0	998.11590	0.055822328
Igsf6	34.0	173.94502	0.055544496
Fcgr3	34.0	1279.76680	0.055811163
Cd86	33.0	2105.79080	0.055710863
Themis2	33.0	415.82825	0.055577688
Fcgr4	33.0	310.27353	0.055500300
Ms4a6c	33.0	146.86868	0.055566620
Cybb	33.0	5207.74660	0.056695793
Ccl6	32.0	927.98834	0.055721990
Clec4n	31.0	164.47343	0.055555556

**Table 2:** The GO_BP terms and pathways enriched for the upregulated genes (A) and the downregulated genes (B) involved in the most significant modules. *GO*, Gene Ontology; *BP*, biological process

Category	Term	Count	*P* value	Gene symbol
(A)				
GO_BP	GO:0002376~immune system process	8	1.63E−07	*C1QA*, *C1QB*, *CD86*, *LY86*, *FCGR1*, *THEMIS2*, *C1QC*, *CLEC4N*
	GO:0045576~mast cell activation	4	3.83E−07	*CD48*, *FYB*, *FCGR2B*, *FCGR3*
	GO:0045087~innate immune response	7	4.62E−06	*C1QA*, *C1QB*, *CYBB*, *LY86*, *FCGR1*, *C1QC*, *CLEC4N*
	GO:0006911~phagocytosis, engulfment	4	1.86E−05	*FCGR2B*, *AIF1*, *FCGR1*, *FCGR3*
	GO:0006954~inflammatory response	5	5.96E−04	*CYBB*, *AIF1*, *LY86*, *THEMIS2*, *CCL6*
PATHWAY	mmu05150:*Staphylococcus aureus* infection	7	2.59E−10	*C1QA*, *C1QB*, *FCGR2B*, *FCGR4*, *FCGR1*, *C1QC*, *FCGR3*
	mmu05322:Systemic lupus erythematosus	6	6.02E−06	*C1QA*, *C1QB*, *CD86*, *FCGR4*, *FCGR1*, *C1QC*
	mmu04145:Phagosome	6	1.37E−05	*CYBB*, *FCGR2B*, *FCGR4*, *CTSS*, *FCGR1*, *FCGR3*
	mmu05152:Tuberculosis	6	1.45E−05	*FCGR2B*, *IL10RA*, *FCGR4*, *CTSS*, *FCGR1*, *FCGR3*
	mmu04380:Osteoclast differentiation	5	8.03E−05	*CYBB*, *FCGR2B*, *FCGR4*, *FCGR1*, *FCGR3*
(B)				
GO_BP	GO:0016125~sterol metabolic process	8	4.60E−14	*CYP51*, *LDLR*, *HMGCR*, *FDPS*, *HMGCS1*, *SREBF2*, *SC4MOL*, *DHCR24*
	GO:0008202~steroid metabolic process	9	5.23E−14	*CYP51*, *LDLR*, *HMGCR*, *FDPS*, *HMGCS1*, *LSS*, *SREBF2*, *SC4MOL*, *DHCR24*
	GO:0008203~cholesterol metabolic process	7	6.80E−12	*CYP51*, *LDLR*, *HMGCR*, *FDPS*, *HMGCS1*, *SREBF2*, *DHCR24*
	GO:0006694~steroid biosynthetic process	7	7.42E−12	*CYP51*, *HMGCR*, *FDPS*, *HMGCS1*, *LSS*, *SC4MOL*, *DHCR24*
	GO:0016126~sterol biosynthetic process	6	1.69E−11	*CYP51*, *HMGCR*, *FDPS*, *HMGCS1*, *SC4MOL*, *DHCR24*
PATHWAY	mmu00100:Steroid biosynthesis	5	1.10E−08	*CYP51*, *SQLE*, *LSS*, *SC4MOL*, *DHCR24*
	mmu00900:Terpenoid backbone biosynthesis	3	2.46E−04	*HMGCR*, *FDPS*, *HMGCS1*

Besides, the PPI network constructed for the downregulated genes included 262 nodes and 503 interactions. In the PPI network, discs, large homolog 4 (DLG4) was the only node with degree larger than 20. Module analysis for the PPI network of the downregulated genes showed that the most significant module (score = 10.364) had 12 nodes and 57 interactions (Fig. 3b). The downregulated genes involved in the most significant module were mainly enriched in sterol metabolic process (GO_BP, *p* value = 4.60E–14) and steroid biosynthesis (pathway, *p* value = 1.10E−08) (Table 2 (B)).

### Integrated network analysis

**Fig. 4:**
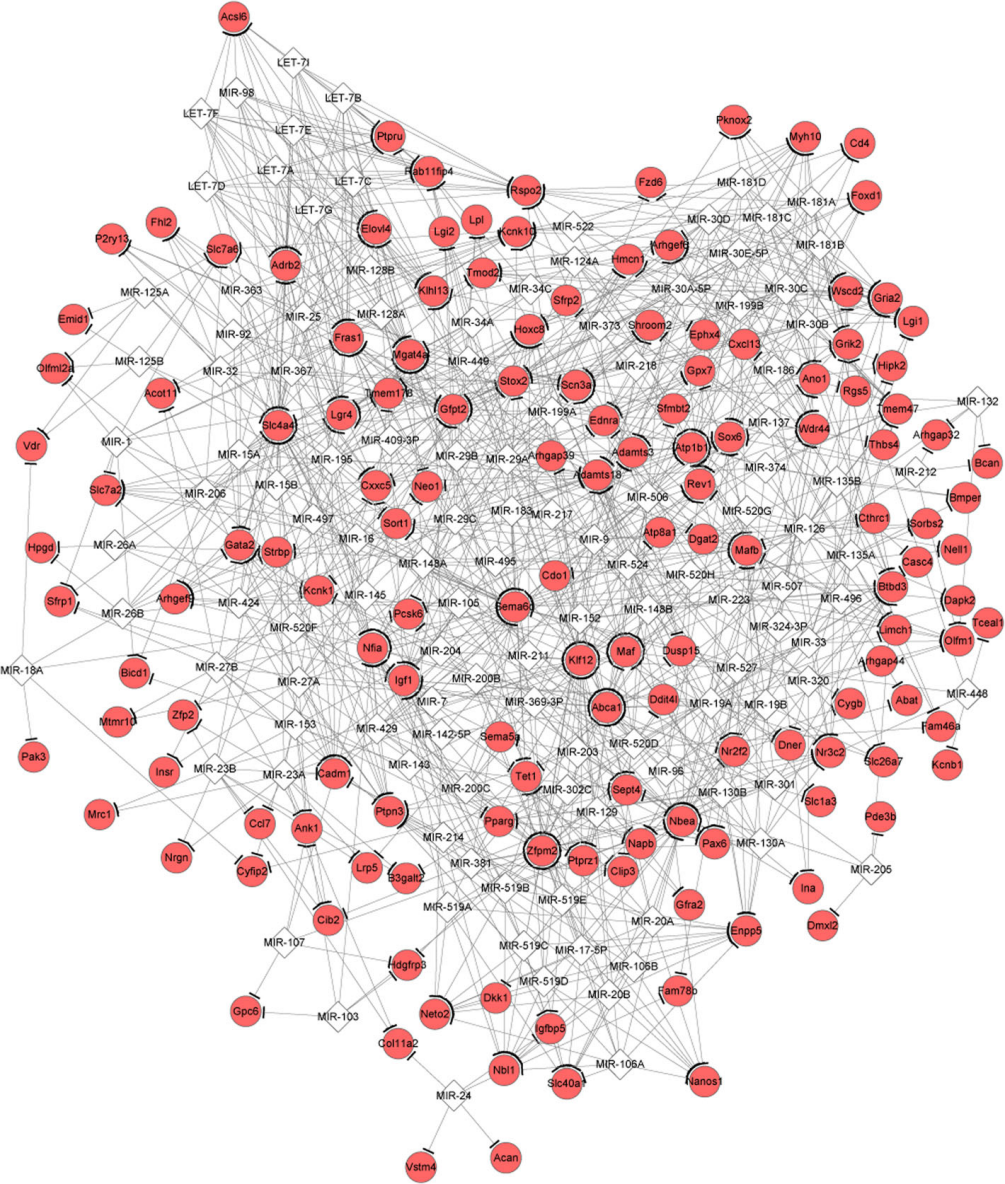
The miRNA-gene regulatory network for the upregulated genes. The interactions of miRNAs-gene were predicted by WEB-based gene set analysis toolkit tool, and regulatory network was visualized using Cytoscape software. Red circles and white quadrangles represent upregulated genes and miRNAs, respectively

**Fig. 5:**
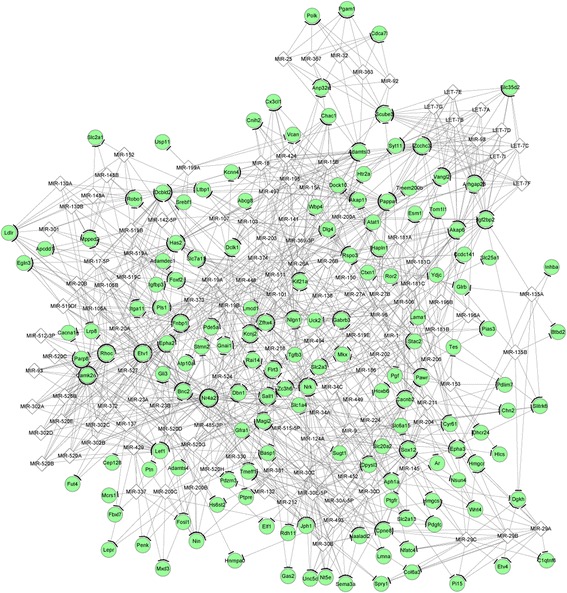
The miRNA-gene regulatory network for the downregulated genes visualized using Cytoscape software. The interactions of miRNAs-gene were predicted by WEB-based gene set analysis toolkit tool, and regulatory network was visualized using Cytoscape software. Green circles and white quadrangles represent downregulated genes and miRNAs, respectively

**Fig. 6:**
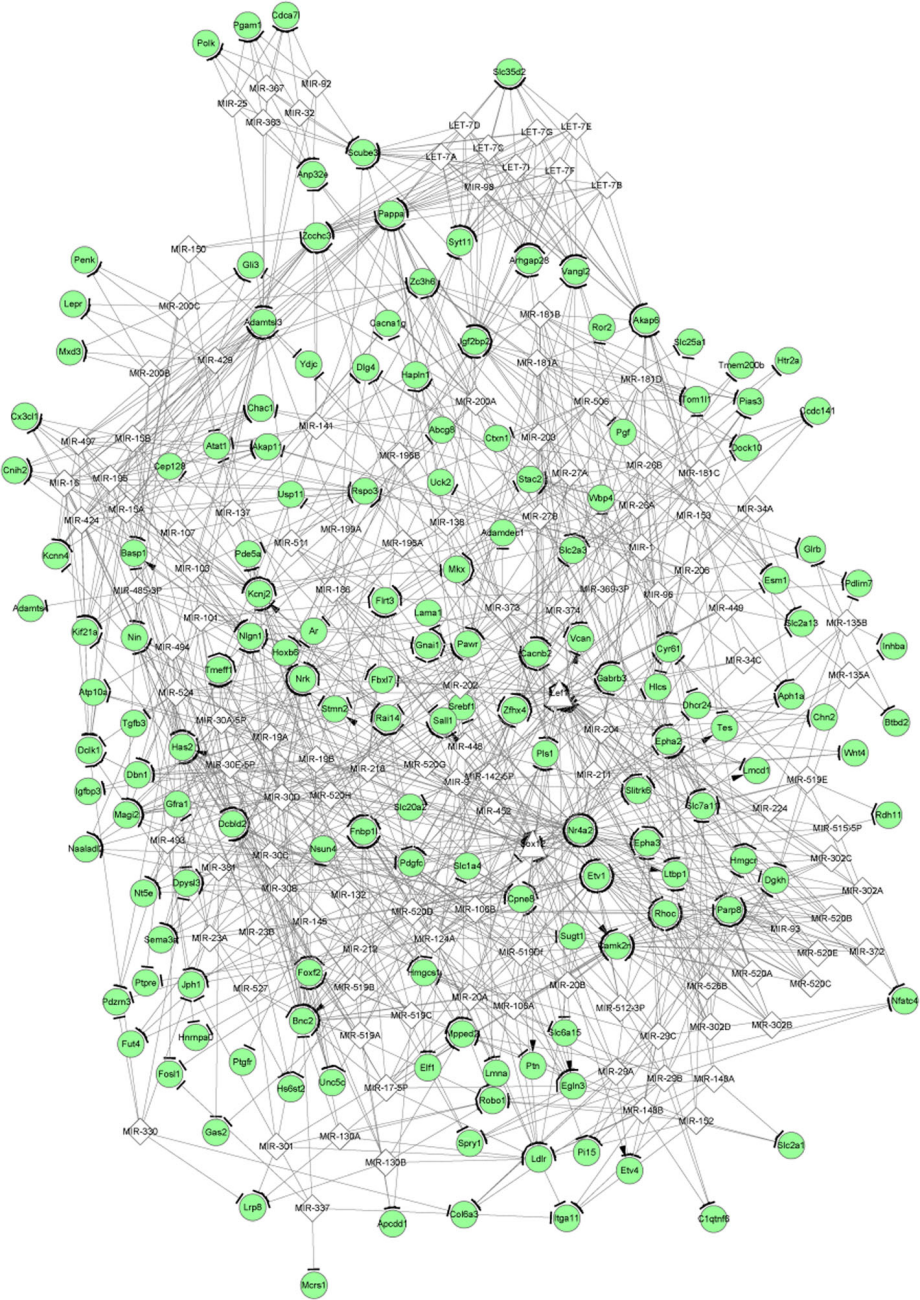
The integrated network for the downregulated genes visualized by Cytoscape software. The miRNA-gene regulatory relationships in the network were predicted by using WEB-based gene set analysis toolkit tool, whereas transcription factor-genes regulatory relationships in the network were predicted by using iRegulon plugin. Green circles, white quadrangles, and white triangles represent downregulated genes, miRNAs, and transcription factors, respectively

The miRNA-DEG regulatory network constructed for the upregulated genes (such as zinc finger protein, multitype 2, *ZFPM2*) and the downregulated genes are separately shown in Figs. 4 and 5. There was no TF predicted for the miRNA-DEG regulatory network of the upregulated genes. Only two TFs (SRY (sex determining region Y)-box 12, *SOX12*, and lymphoid enhancer binding factor 1, *LEF1*) were predicted for the miRNA-DEG regulatory network of the downregulated genes. The integrated network for the downregulated genes had 278 nodes and 1144 edges (Fig. 6). Especially, *LEF1*, nuclear receptor subfamily 4 group A member 2 (*NR4A2*), hyaluronan synthase 2 (*HAS2*), and ras homolog family member C (*RHOC*) had higher degrees in the integrated network.

## Discussion

In this study, a total of 871 DEGs were identified in the PTHR1 knockdown OS samples compared with the control OS samples, including 438 upregulated and 433 downregulated genes. *ZFPM2* was involved in the miRNA-DEG regulatory network constructed for the upregulated genes. There was no TF predicted for the miRNA-DEG regulatory network of the upregulated genes. Besides, TF *LEF1* was predicted for the miRNA-DEG regulatory network of the downregulated genes. In the integrated network, *LEF1*, *NR4A2*, *HAS2*, and *RHOC* had higher degrees.

In rat bone marrow stromal cells (BMSCs), neurotrophic factors, *NURR1* (also named *NR4A2*), tyrosine hydroxylase (TH), and nestin genes have spontaneous expression [26]. *NURR1* and *NURR77* may contribute to increase the migratory potential of fetal FBMSCs, which may mediate the local immune response specifically [27]. *NURR1* and PPARγ coactivator-1α (*PGC-1α*) may play pivotal roles in mediating osteoblast function and cAMP-dependent osteoblast gene expression [28, 29]. *NURR1* maintains cartilage homeostasis via selectively inhibiting the expression of *MMP* gene during inflammation [30]. These declared that *PTHR1* might function in OS through targeting *NR4A2*.

Hyaluronan synthesized by *HAS2* affects the proliferation, invasion, and motility of MG-63 OS cells [31]. Through regulating the expressions of versican, *HAS2*, and hyaluronan, transforming growth factor β2 (*TGF-β2*) may lead to the metastasis of OS cells [32]. *HAS2* plays critical roles in osteoblast differentiation and development by mediating high molecular weight hyaluronan synthesis [33]. *RHOC* and *MMP9* expression levels have close association, and their high expressions are closely correlated with the formation, development, invasion, and metastasis of OS [34]. *RHOC* has different expressions in SOSP-9607E10 and SOSP-9607H9 OS cell lines, and its overexpression functions in the invasion and metastasis of OS through inducing cell migration [35]. Therefore, *HAS2* and *RHOC* might also be targets of *PTHR1* in OS.

Via regulating the expression of *ZFPM2*, hypoxia-induced *miR-429* contributes to the differentiation of osteoblastic cells [36]. *LEF1* can delay osteoblast maturation and regulates the expression levels of some genes in osteoblasts [37]. *LEF1* has an essential role in the activation of alpha 1 (XI) collagen (*COL11A1*), and *COL11A1* inhibits the terminal differentiation of osteoblasts [38]. *LEF1* serves as a transcriptional effector of the Wnt/β-catenin pathway and is critical for the tumor invasion induced by hepatocyte growth factor (*HGF*) [39]. *LEF1* mediates bone density, osteoblast differentiation, and skeletal strength, and *Lef1ΔN* regulates the terminal differentiation in osseous cells [40, 41]. Thus, *ZFPM2* and *LEF1* might be targets of PTHR1 in OS.

## Conclusions

In conclusion, a total of 871 DEGs were screened from the *PTHR1* knockdown OS samples. Besides, *ZFPM2*, *LEF1*, *NR4A2*, *HAS2*, and *RHOC* might be targets of *PTHR1* in OS. However, more experimental researches should be conducted to confirm these findings obtained from bioinformatics analysis.

## Abbreviations

*ACK1*: Activated Cdc42-associated kinase
*AEG1*: Elevated gene 1
BC: Betweenness centrality
BMSCs: Bone marrow stromal cells
BP: Biological process
CCo: Cellular component
CCe: Closeness centrality
DAVID: Database for Annotation, Visualization and Integrated Discovery
DC: Degree centrality
DEGs: Differentially expressed genes
DLG4: Discs, large homolog 4
FC: Fold change
FDR: False discovery rate
GEO: Gene Expression Omnibus
GO: Gene Ontology
*HAS2*: Hyaluronan synthase 2
*HGF*: Hepatocyte growth factor
*ING4*: Inhibitor of growth 4
KEGG: Kyoto Encyclopedia of Genes and Genomes
MF: Molecular function
*MMP2*: Matrix metalloproteinase 2
NF-κB: Nuclear factor kappa B
*NR4A2*: Nuclear receptor subfamily 4 group A member 2
OS: Osteosarcoma
*PGC-1α*: PPARγ coactivator-1α
PPI: Protein-protein interaction
PTHR1: Parathyroid hormone receptor 1
*RHOC*: Ras homolog family member C
RMA: Robust Multiarray Average
STRING: Search Tool for the Retrieval of Interacting Genes
TFs: Transcription factors
*TGF-β2*: Growth factor β2
TH: Neurotrophic factors, tyrosine hydroxylase.
